# A Case of Solid Pseudopapillary Tumor of the Pancreas

**DOI:** 10.7759/cureus.45399

**Published:** 2023-09-17

**Authors:** Albert Tine, Heong Jin C Ahn, Arsh N Patel, William Laidig, Nathan Zhang, Todd J Kendall, Latasha Henry

**Affiliations:** 1 Department of Research, Alabama College of Osteopathic Medicine, Dothan, USA; 2 Pathology, Ascension Providence Hospital, Mobile, USA; 3 Gastroenterology, Ascension Providence Hospital, Mobile, USA

**Keywords:** pancreas, pancreatic neoplasms, abdominal pain, pancreas disease, solid pseudopapillary tumor

## Abstract

We report a rare case of solid pseudopapillary neoplasm in a 24-year-old woman, who presented with progressively worsening left epigastric and right lower quadrant abdominal pain for several weeks. A CT scan showed a mass in the tail of the pancreas that extended to the hilum of the spleen. Endoscopic ultrasound (EUS)-guided fine needle aspiration (FNA) and immunohistochemical analysis exhibited findings pathognomonic for solid pseudopapillary neoplasm. The patient underwent distal pancreatectomy and splenectomy. Post-surgical biopsy confirmed the FNA findings, with the tumor confined to the pancreas and no extension to nearby structures. The patient did not require any other adjuvant therapy. She was asymptomatic at the one-month follow-up and showed no signs of disease. We discuss the unique circumstances of this case and highlight the importance of differentiating this tumor from other pancreatic neoplasms.

## Introduction

Solid pseudopapillary tumor (SPT) of the pancreas, also known as Frantz tumor, is a rare type of neoplasm that primarily affects the pancreas [1]. It accounts for approximately 1-3% percent of all pancreatic tumors. It is more commonly observed in young females, typically in their second or third decade of life, although it can occur in individuals of any age or gender. SPTs are typically well-encapsulated, solitary masses with a mixture of solid and cystic components. The term "pseudopapillary" refers to the tumor's distinctive histological appearance, characterized by papillary projections and solid areas. These tumors have low malignant potential, with a favorable prognosis compared to other pancreatic malignancies. SPTs are frequently asymptomatic or minimally symptomatic. However, when symptoms do occur, they can include abdominal pain or discomfort, a palpable mass in the abdomen, nausea, vomiting, or loss of appetite.

The exact histopathogenesis of the tumor cells within SPT remains uncertain and is still a matter of debate. Several theories suggest that SPT may arise from various cell types within the pancreas, such as acinar cells, endocrine cells, ductal cells, or pancreatic progenitor cells [1,2]. The exact cell of origin is yet to be definitively determined. SPTs can be visualized using various imaging modalities, including CT, ultrasonography (US), and MRI. These imaging techniques can help differentiate SPT from other pancreatic lesions such as mucinous cystadenoma or pancreatic pseudocyst and provide valuable information about the tumor's size, location, and characteristics. The primary treatment for SPT involves complete surgical resection, which is often curative, resulting in an excellent prognosis for the majority of patients. However, there have been reports of fatal cases of metastasizing SPTs of the pancreas, which suggests that these tumors can exhibit aggressive clinical behavior [2]. In this report, we present a case of SPT in a young African American female with a benign clinical course.

## Case presentation

A 24-year-old African American woman with no significant past medical history presented with complaints of left mid and right lower quadrant abdominal pain. The pain had first occurred several weeks prior and was intermittent. She described the pain as twisting in nature and radiating to the epigastric region and it was getting progressively worse. It was associated with nausea without vomiting or early satiety. She reported intermittent subjective fever for several weeks. Oral intake was an aggravating factor. A review of systems was otherwise unremarkable. Her vital signs were stable. On physical examination, she was anicteric, her abdomen was tender to palpation in the left epigastric and right lower quadrant but otherwise soft and non-distended, and there was no rebounding or guarding sign. There were no palpable masses and bowel sounds were heard in all four quadrants. The key laboratory findings were within normal limits (Table 1).

**Table 1:** Key laboratory findings

Lab	Patient value	Normal value
White blood cells	7.5 x 10^9^/L	4.5-11 x 10^9^/L
Neutrophils	45.3%	40-60%
Alkaline phosphatase (ALP)	83 IU/L	44-147 IU/L
Alanine transaminase (ALT)	13 IU/L	7-55 IU/L
Aspartate transaminase (AST)	16 IU/L	8-33 IU/L
Total bilirubin	0.3 mg/dL	0.2-1.3 mg/dL
Amylase	46 IU/L	40-140 IU/L
Lipase	51 IU/L	0-160 IU/L
CA 19-9	12 U/mL	0-37 U/mL

**Figure 1:**
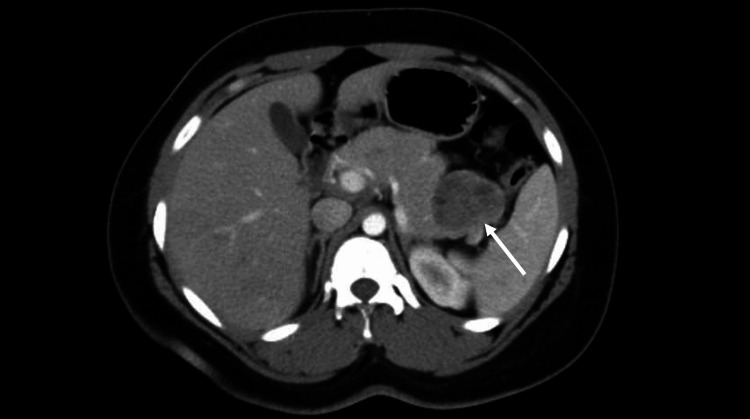
CT abdomen and pelvis with contrast exhibiting a 3.3 cm pancreatic mass adjacent to splenic hilum (white arrow) CT: computed tomography

**Figure 2:**
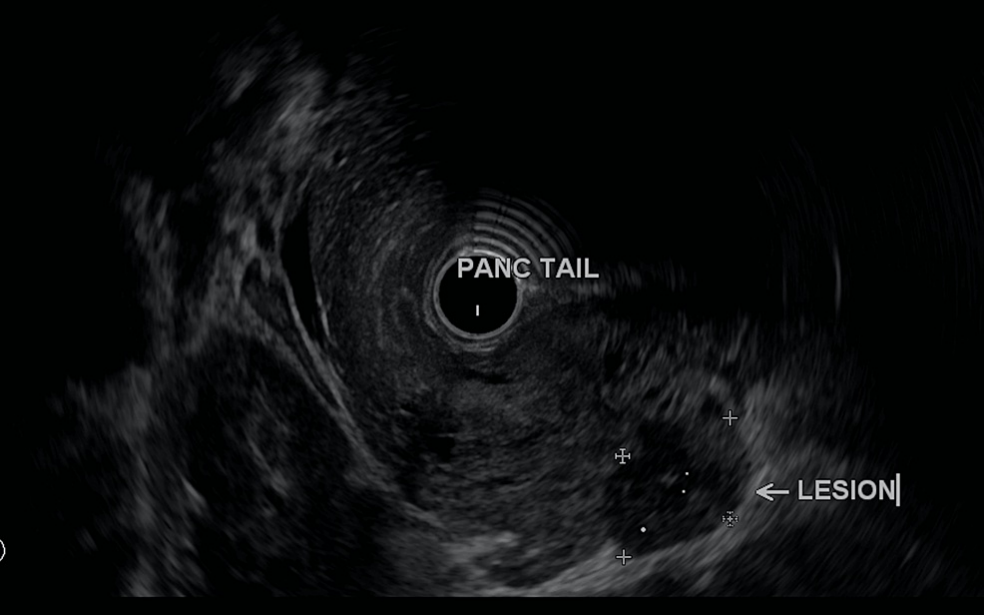
EUS exhibiting a hypoechoic 3.3 cm mass at the pancreatic tail (white arrow) EUS: endoscopic ultrasound

CT scan of the abdomen and pelvis with iodinated contrast (Figure 1) displayed a 3.3 x 4.7 cm mass arising from the tail of the pancreas. Esophagogastroduodenoscopy (EGD) with endoscopic ultrasound (EUS) confirmed the presence of a well-circumscribed hyperechoic pancreatic tail mass with areas of hypoechogenicity and no definite vascular invasion (Figure 2). The common bile duct (CBD) was non-dilated at 5.2 mm (<6-7 mm). The pancreatic duct (PD) was non-dilated with the head measuring 2.8 mm (<3 mm), body 1.3 mm (<2 mm), and tail 0.6 mm (<1 mm). Fine needle aspiration (FNA) showed tumor cells with papillary architecture. An immunohistochemical analysis revealed that the tumor cells were positive for progesterone receptor, pankeratin, CD10, and beta-catenin. These findings were pathognomonic of SPT.

**Figure 3:**
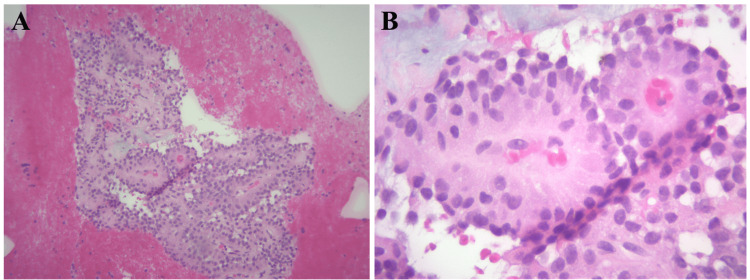
Histopathology exhibiting pseudopapillary cores (A) 10x magnification; (B) 40x magnification

The patient underwent a laparoscopic resection of the body/tail of the pancreas and splenectomy for SPT. The postoperative recovery was complicated by pancreatic leakage, marked by persistent elevation of amylase in the 19 French JP drain, and poorly controlled postoperative pain. She also had minor leukocytosis postoperatively, which improved with empiric antibiotics, and she was discharged on postop day seven. Histological examination demonstrated a solid and vascular pattern with papillary-like architecture (Figure 3). The tumor was confined to the pancreas with no lymph-vascular and perineural invasion. The margins were negative. The patient has been asymptomatic since the procedure and has not required any adjuvant therapy. The follow-up baseline CT scans showed no residual disease.

## Discussion

SPT of the pancreas is a rare tumor of the exocrine pancreas and accounts for approximately 1-3% of all pancreatic tumors. Initially described by Virginia Frantz in 1959, this tumor had been referred to by various names until it was officially designated as "solid pseudopapillary tumor" of the pancreas by the World Health Organization (WHO) in 1996 [1,2]. The exact histogenesis of this tumor remains unclear. There are many varying theories about its origin. These include a neuroendocrine origin, an exocrine centroacinar cell origin, a ductal epithelial cell origin, an extrapancreatic origin from genital ridge/ovarian anlage cells, or possibly a totipotent primordial cell origin that would further differentiate into other cell types [3-5]. This rare tumor seems to occur most commonly in the second to third decades of life among non-Caucasian women, specifically Asian and African Americans. Most patients with SPT exhibit minimal or no symptoms with a significant number of cases being incidentally discovered. The clinical presentation is usually nonspecific with possible symptoms including abdominal pain, weight loss, vomiting, or the presence of an abdominal mass [4].

While most cases of SPT usually run a benign clinical course, some large-scale studies have demonstrated malignancy through local recurrences or metastasis. Reported studies suggest that the most common locations of metastasis are the liver and omentum [1,6-7]. However, in very rare cases, such as our patient, there have been reports of metastasis to the spleen or accessory splenic structures [4-5]. Furthermore, SPTs that are located in the head of the pancreas seem to demonstrate significantly lower survivability due proximity of other significant structures than those in the more common body/tail regions [3]. The morphological characteristics of SPT can range from solid to cystic components accompanied by cellular degenerative changes [1,8]. Immunohistochemical staining generally reveals characteristic positivity for beta-catenin, vimentin, cyclin D1, α1-antitrypsin, CD56, and CD10 [3,5]. Also, LEF1 is a more commonly used stain nowadays, and it has been found to be positive within these tumors as well [5]. Most tumors are diagnosed through abdominal ultrasound or CT scan with confirmation done through EUS biopsy [1,5].

Histologically, this case exhibited a highly characteristic appearance, with neoplastic cells displaying a uniform arrangement surrounding small vessels, resembling papillary structures. This distinctive pattern is commonly referred to as the "pseudopapillary pattern”. Another common characteristic histological aspect of SPT is the formation of cysts through extensive hemorrhage and necrosis [2]. SPTs possess a low-grade malignant potential and typically exhibit a good prognosis, even when metastatic disease is present. Patients who undergo surgical resection have a reported five-year survival rate of up to 97% [1,9]. Since these tumors are usually localized, surgery is usually the first-line treatment [10]. In contrast to the majority of SPT cases, our patient’s treatment plan warranted a splenectomy due to metastasis.

## Conclusions

We discussed a case of SPT of the pancreas in a young African American female. The tumor manifested as a sizable mass located in the pancreatic body/tail with an extension to the hilum of the spleen. The patient underwent a successful laparoscopic distal pancreatectomy and splenectomy and had a favorable postoperative recovery. Despite the histopathological analysis suggestive of potential aggressiveness, the patient exhibited no signs of recurrent disease at the three-, six-, and 12-month follow-up appointments.
